# *Penicillium chrysogenum* as a model system for studying cellular effects of methylglyoxal

**DOI:** 10.1186/s12866-015-0472-y

**Published:** 2015-07-10

**Authors:** Christian Q. Scheckhuber

**Affiliations:** Senckenberg Research Institute, LOEWE Excellence Cluster for Integrative Fungal Research (IPF), Georg-Voigt-Str. 14-16, D-60325 Frankfurt am Main, Germany

**Keywords:** Autophagy, Green-fluorescent protein, Methylglyoxal, Mitochondria, *Penicillium chrysogenum*, Peroxisomes, Stationary phase

## Abstract

**Background:**

α-oxoaldehydes are formed as toxic by-products during metabolic activity. The biologically most important compound of this class, methylglyoxal, results from spontaneous phosphate elimination from dihydroxyacetone phosphate and glyceraldehyde 3-phosphate which are intermediate glycolysis products. Methylglyoxal-mediated modification of lipids, nucleic acids and proteins is known to lead to the formation of advanced glycation end products. These modifications contribute to the aetiology of severe diseases like diabetes and neurodegenerative disorders. By using simple model organisms it is possible to conveniently study the effects of methylglyoxal on cellular processes. Here, results are presented on the effects of methylglyoxal on mycelium growth, stationary phase entry (monitored by autophagy induction), mitochondrial morphology and protein composition in the filamentous fungus *Penicillium chrysogenum*.

**Results:**

Methylglyoxal leads to growth rate reduction of this fungus so that the entry into the stationary phase is delayed. Mitochondrial morphology is not changed by methylglyoxal. However, rapamycin-mediated fragmentation of mitochondria is prevented by methylglyoxal. Furthermore, three proteins are identified that are present in lower abundance when methylglyoxal is added to the growth medium (aldo-keto reductase [Pc22g04850], 5-methyl-tetrahydropteroyl-triglutamate-homocysteine S-methyltransferase [Pc22g18630] and NAD-dependent formate dehydrogenase [Pc12g04310]).

**Conclusions:**

The presented results contribute to the understanding of cellular pathways and mechanisms that are affected by the ubiquitous α-oxoaldehyde methylglyoxal.

## Background

Living cells depend on the enzymatic conversion of D-glucose to pyruvate via glycolysis for the formation of the molecular energy stores adenosine triphosphate (ATP) and nicotinamide adenine dinucleotide (NADH). Glycolysis, however, can also lead to the production of toxic by-products. The best studied and probably the most relevant compound of this class is the α-oxoaldehyde methylglyoxal (also known as pyruvaldehyde or 2-oxopropanal) [1]. Methylglyoxal is mainly formed by spontaneous elimination of phosphate from the glycolysis intermediates dihydroxyacetone phosphate (DHAP) and, to a lesser extent, glyceraldehyde 3-phosphate [2]. Another metabolic route that leads to the formation of methylglyoxal is the oxidation of aminoacetone in the catabolism of L-threonine which is mediated by the semicarbazide-sensitive amine oxidase [3]. Methylglyoxal can also be formed as a side-product of lipid peroxidation under conditions of elevated intracellular oxidative stress [4]. This α-oxoaldehyde causes protein glycation and functional impairments due to its high reactivity with amino groups of proteinaceous lysine and arginine residues [5]. These residues are modified further and ultimately converted into ‘advanced glycation end-products’ (AGEs). For example, a major type of AGE in cells of the human eye lens from aged donors is the hydroimidazolone N^ε^-(5-hydro-5-methyl-4-imidazolon-2-yl)-ornithine (MG-H1) [6]. Importantly, the MG-H1 modification is found to be also increased in pathological conditions like Alzheimer’s disease, arthritis, diabetes, renal failure and Parkinson’s disease [7]. Collagen type IV [8] and hemoglobin [9, 10] in humans and enolase 2 in baker’s yeast [11] have been identified as main targets of methylglyoxal-mediated modifications. Collagen type IV loses its ability to interact with integrins whereas the function of haemoglobin is compromised by an intensified oxygen binding capacity. For yeast enolase 2 studies have been performed in which *in vivo* and *in vitro* glycation patterns after methylglyoxal treatment were compared. It was revealed that *in vivo* glycation of enolase 2 is a specific process where certain lysine and arginine amino acids are consistently modified.

Unsurprisingly, cells possess protective mechanisms that prevent or limit glycation damage. The best characterised enzymatic system that detoxifies methylglyoxal by conversion to D-lactate consists of three components: glyoxalase I (GLO1, lactoylglutathione lyase), glyoxalase II (GLO2, hydroxyacylglutathione hydrolase) and catalytic amounts of reduced glutathione [12]. One example demonstrating the importance of the glyoxalase system for protecting organisms from the deleterious effects of α-oxoaldehydes and subsequent glycation stems from work on nematodes. Overexpression of the *Glo1* gene in *Caenorhabditis elegans* was found to increase the lifespan of this organism by protecting components of the mitochondrial respiratory chain from glycation [13]. In addition to glyoxalase I and glyoxalase II a novel type of glyoxalase has been reported that is working independently of glutathione [14]. Mutant variants of this enzyme (DJ-1/PARK7) have been reported to contribute to the aetiology of autosomal recessive early-onset Parkinsonism [15]. Later studies revealed that some of these mutations affect mitochondrial integrity in cellular models of Parkinsonism [16, 17]. Recently, it was demonstrated that D-lactate, which is also formed by DJ-1, is able to positively influence mitochondrial function by maintaining the membrane potential of these organelles [18]. Collectively these results demonstrate that not only the removal of methylglyoxal but also an end-product of glyoxalase activity is important for maintaining cellular health and integrity. Other enzymes that may limit the formation of methylglyoxal are methylglyoxal reductase [19], aldo-keto reductases [20], α-oxoaldehyde dehydrogenase and betaine dehydrogenase [21] although their contribution to methylglyoal degradation has been investigated less.

Recently the role of methylglyoxal and its degradation by the glyoxalase system on fungal senescence was studied in the filamentous ascomycete *Podospora anserina* [22]. It was shown that overexpression of genes encoding glyoxalase I (*PaGlo1*) and glyoxalase II (*PaGlo2*) has a beneficial effect on viability and delayed the onset of senescence in this model system while the deletion of *PaGlo1* resulted in a strongly decreased lifespan compared to the wild type when the strains were grown in the presence of 2 % (w/v) glucose which is supposed to increase the formation of methylglyoxal. These results suggest a link between intracellular methylglyoxal levels and fungal viability. Subsequently, the impact of increased glyoxalase I and II levels was studied in an industrial producer of β-lactam antibiotics, *Penicillium chrysogenum* [23]. Overexpression of *PcGlo1* and *PcGlo2* led to the improvement of penicillin (PEN) production in this fungus. Analysis of protein levels revealed that increased levels of two enzymes of the biosynthetic pathway for PEN are present in the *PcGlo1*/*PcGlo2* overexpression strain. One of these enzymes, isopenicillin-N acyltransferase (IAT), is localised to peroxisomes [24], organelles of anti-oxidative activity and sites of various pathways of secondary metabolism.

The aim of the present study was to elucidate the effects of exogenous methylglyoxal on mycelium growth, initiation of the stationary phase, morphology of mitochondria and overall protein composition in *P. chrysogenum*. It is revealed that methylglyoxal affects the growth rate of the mycelium so that initiation of the stationary phase is delayed. Methylglyoxal has no effect on mitochondrial morphology: thread-like mitochondria are present in mycelia independent of methylglyoxal treatment. By using differential isoelectric focusing (IEF)/two-dimensional sodium dodecyl sulphate polyacrylamide electrophoresis (2D-SDS-PAGE) in combination with peptide mass fingerprinting three proteins that are present in lower abundance compared to the control when methylglyoxal is added to the growth medium are identified.

## Results

### Effects of methylglyoxal on mycelium growth

**Fig. 1:**
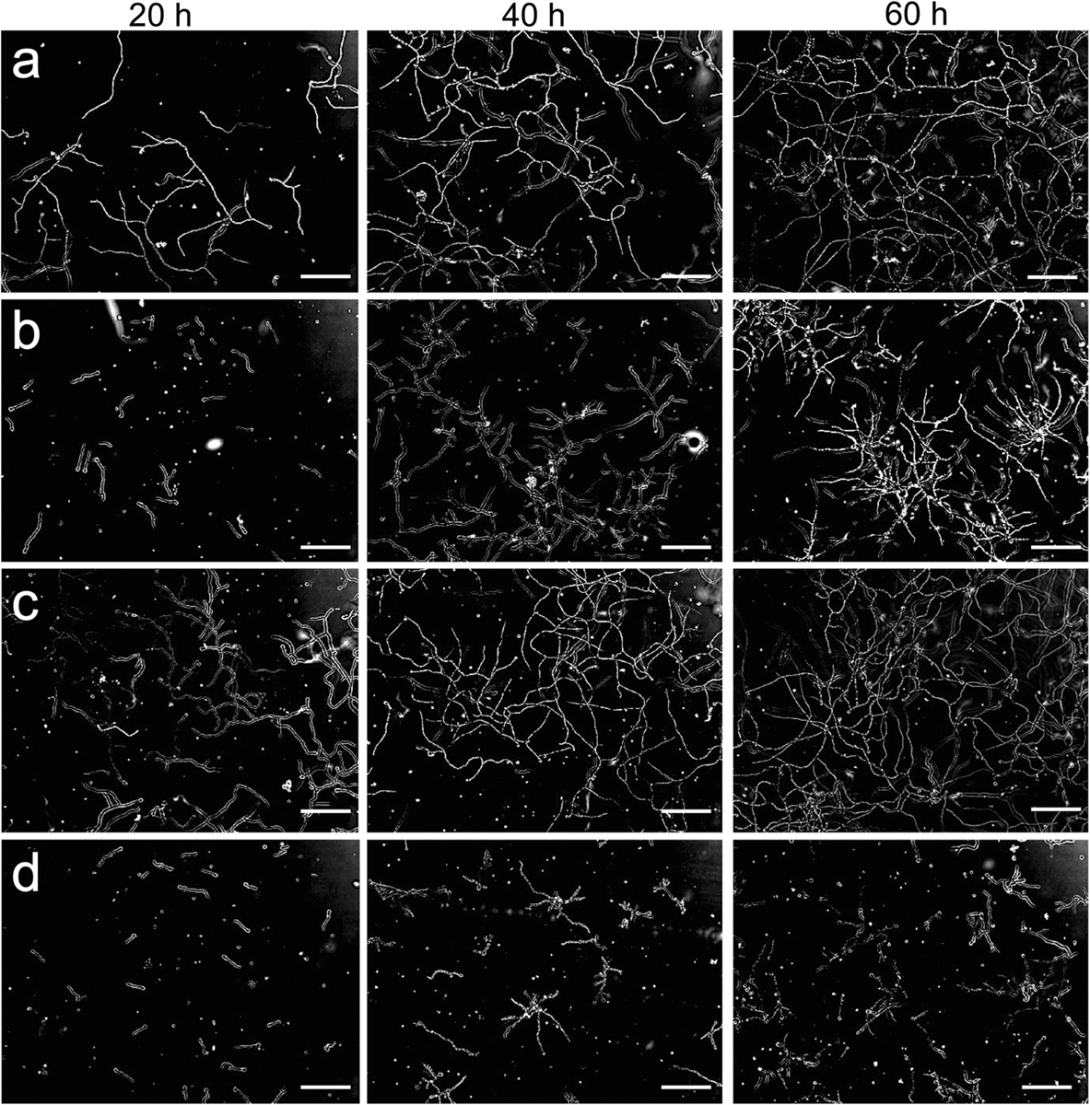
Effects of methylglyoxal and rapamycin on growth of *P. chrysogenum* Ws54-1255 (GFP-SKL). At the indicated times pictures from cultures grown on starvation pads were taken at the indicated time points. Representative images are shown for each time point. **a** control; **b** + 0.05 % (v/v) methylglyoxal; **c** + 1 μM rapamycin; **d** + 0.05 % (v/v) methylglyoxal/+ 1 μM rapamycin. Scale bars: 100 μm

Although the consequences of overexpression of the glyoxalase-encoding genes *PcGlo1* and *PcGlo2* for penicillin production have recently been studied [23] the effects of exogenously added methylglyoxal on the growth behaviour of *P. chrysogenum* have so far not been investigated. The *P. chrysogenum* strain Ws54-1255 (GFP-SKL) was grown on agarose pads without added nutrients, ‘starvation pads’ [25], in the presence or absence of exogenously added methylglyoxal and analysed by light microscopy (Fig. 1). A methylglyoxal concentration of 0.05 % (v/v) was found to be suited for the studies because lower concentrations (e. g., 0.005 % [v/v] and 0.01 % [v/v]) cause no observable changes on the growth behaviour and higher concentrations (e. g., 0.1 % [v/v]) led to almost complete inhibition of growth. The untreated control shows pronounced growth after 20 h and 40 h of incubation (Fig. 1a). At 60 h roughly the same amount of mycelium is present compared to 40 h which is an indication for a stop of cell growth. This is probably based on nutrient limitation encountered by the mycelium after prolonged growth on the starvation pads. The samples subjected to 0.05 % (v/v) methylglyoxal grow initially (20 h) much more slowly compared to the control (Fig. 1b). This retardation of growth demonstrates that methylglyoxal can enter the fungal cells and interfere with their function. At later time points (40 h and 60 h) the colonies are still growing although their morphology looks quite irregular (Fig. 1b). In samples treated with 1 μM rapamycin for maximum induction of autophagy mycelium growth stalls (similar to the untreated control) at 40 h (Fig. 1c). Rapamycin is a well-known inhibitor of TOR (‘target of rapamycin’) signalling [26]. This treatment leads to pronounced induction of autophagy in most organisms [27]. When rapamycin and methylglyoxal are added in combination mycelium growth at all time points is reduced even further compared to samples subjected solely to methylglyoxal (Fig. 1d). Therefore rapamycin treatment seems to sensitize *P. chrysogenum* towards exogenously added methylglyoxal.

**Fig. 2:**
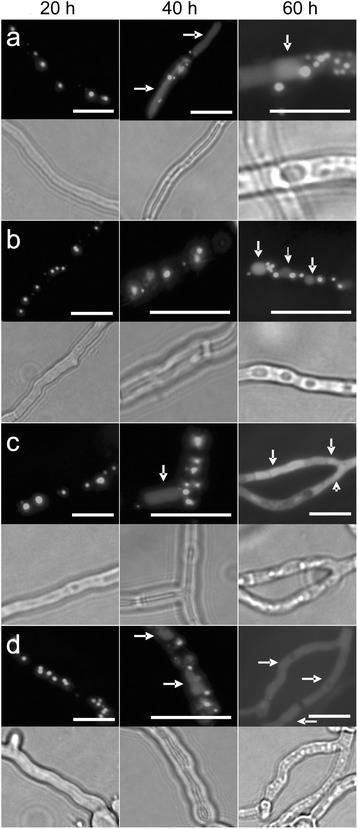
Localisation of GFP-SKL in Ws54-1255 (GFP-SKL) treated with methylglyoxal and/or rapamycin. At the indicated times cultures grown on starvation pads supplemented with or without methylglyoxal and/or rapamycin were analysed using fluorescence microscopy. Representative images are shown for each time point. **a** control; **b** + 0.05 % (v/v) methylglyoxal; **c** + 1 μM rapamycin; **d** + 0.05 % (v/v) methylglyoxal/+ 1 μM rapamycin. Corresponding bright field areas are shown below each fluorescence channel image. White arrows: GFP-SKL localised to vacuoles. Scale bars: 10 μm

The observed differences in mycelium growth in the presence or absence of methylglyoxal could be accompanied by a delayed entry into stationary phase. In filamentous fungi, autophagy induction is a marker for stationary phase entry. Therefore degradation of GFP-SKL labelled peroxisomes as an autophagy marker was analysed by fluorescence microscopy (Fig. 2, Table 1). The utilised assay is based on the principle that GFP is not efficiently degraded by proteases localized in vacuoles [28]. Therefore the presence of GFP in vacuoles is a measure for peroxisomal degradation in the Ws54-1255 (GFP-SKL) reporter strain. In untreated samples vacuoles filled with GFP become visible after 40 h of incubation and these are also present after 60 h (Fig. 2a). At the later time point GFP localized to the vacuole lumen is observed in ca. 50 % of hyphae (Table 1). By contrast, in samples subjected to 0.05 % (v/v) methylglyoxal no GFP-labelled vacuoles are observed up to 40 h (Fig. 2b, Table 1) indicating that these mycelia have not entered stationary phase by this time point. At a later time point (60 h) GFP-labelled vacuoles become visible (Fig. 2b) but only in approximately 25 % of hyphae (Table 1). After 40 h pronounced vacuolation of hyphae is observed when 1 μM rapamycin was added to the samples (Fig. 2c, Table 1). These vacuoles contain GFP suggesting peroxisomal degradation by autophagy (pexophagy). At 60 h almost all hyphae contain GFP-labelled vacuoles (Table 1) which are differing in morphology from vacuoles observed at 40 h (their structure seems less regular and they are filled with granulate matter which might be due to the high level of degradation occurring in strains that are subjected to both starvation and rapamycin). When rapamycin and methylglyoxal are synergistically applied the following observations are made (Fig. 2d): (i) at 20 h, there is no apparent difference to the untreated control and exclusive methylglyoxal treatment, (ii) at 40 h a low level of peroxisome degradation is detected which is not seen in hyphae treated solely with methylglyoxal and (iii) at 60 h the tendency of GFP to localise to vacuoles is similar to samples treated with methylglyoxal alone. The phenotype at 40 h indicates that methylglyoxal cannot halt the induction of peroxisome degradation by rapamycin treatment.

**Table 1:** Analysis of autophagy in control (Ws54-1255 (GFP-SKL)) and Δ*atg1* (GFP-SKL) strains

	20 h	40 h	60 h
Control	-	+	++
+ MG	-	-	+
+ R	-	++	+++
+ MG/+ R	-	(+)	+
Δ*atg1*	-	-	-
+ MG	-	-	-
+ R	-	-	-
+ MG/+ R	-	-	-

For each data point around 100 hyphae were analysed by fluorescence microscopy. -: no peroxisome degradation observed, (+): weak peroxisome degradation (5–10 % of all hyphae), +: ca. 25 % of hyphae display peroxisome degradation, ++: ca. 50 % of hyphae display peroxisome degradation, +++: 90–100 % of hyphae display peroxisome degradation.*MG* methylglyoxal (0.05 % [v/v]). *R* rapamycin (1 μM)

**Fig. 3:**
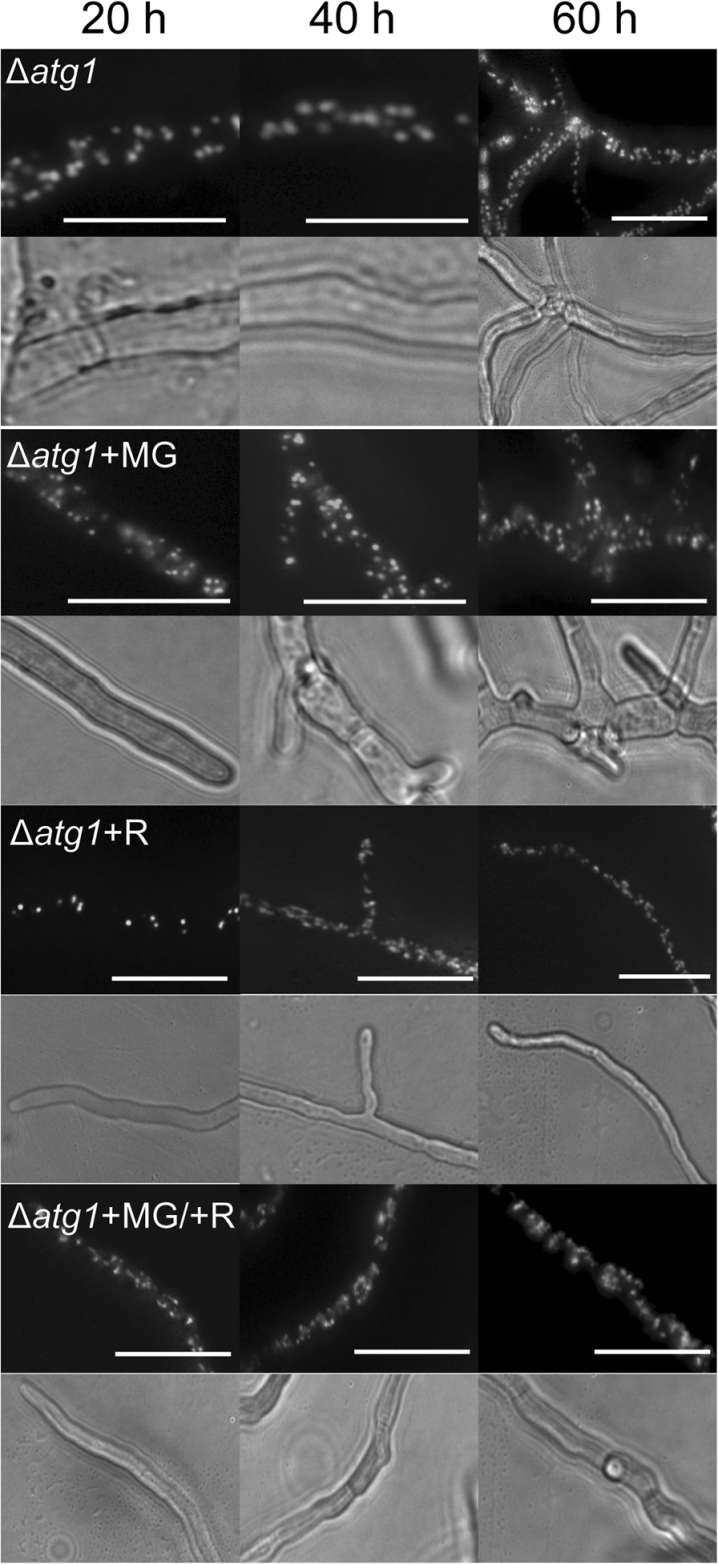
Localisation of GFP-SKL in *P. chrysogenum* Δ*atg1* (GFP-SKL) treated with methylglyoxal and/or rapamycin. At the indicated times cultures grown on starvation pads were analysed using fluorescence microscopy. Representative images are shown for each time point. Corresponding bright field areas are shown below each fluorescence channel image. + MG: 0.05 % (v/v); + R: 1 μM rapamycin. Scale bars: 10 μm

As negative control for autophagy induction the Δ*atg1* (GFP-SKL) strain was utilized (Fig. 3) which is defective in both bulk (unselective) and selective autophagy [29]. This strain never shows a vacuolar localisation of GFP regardless whether there is methylglyoxal present in the growth medium or not (Fig. 3, Table 1). There are more peroxisomes present in Δ*atg1* (GFP-SKL) compared to Ws54-1255 (GFP-SKL), an effect that has been previously reported [29]. These findings suggest that GFP-SKL is not transported to vacuoles by autophagy-independent processes.

Collectively the presented data suggest that methylglyoxal affects mycelium growth by delaying the entry into stationary phase.

### Effects of methylglyoxal on mitochondrial morphology

**Fig. 4:**
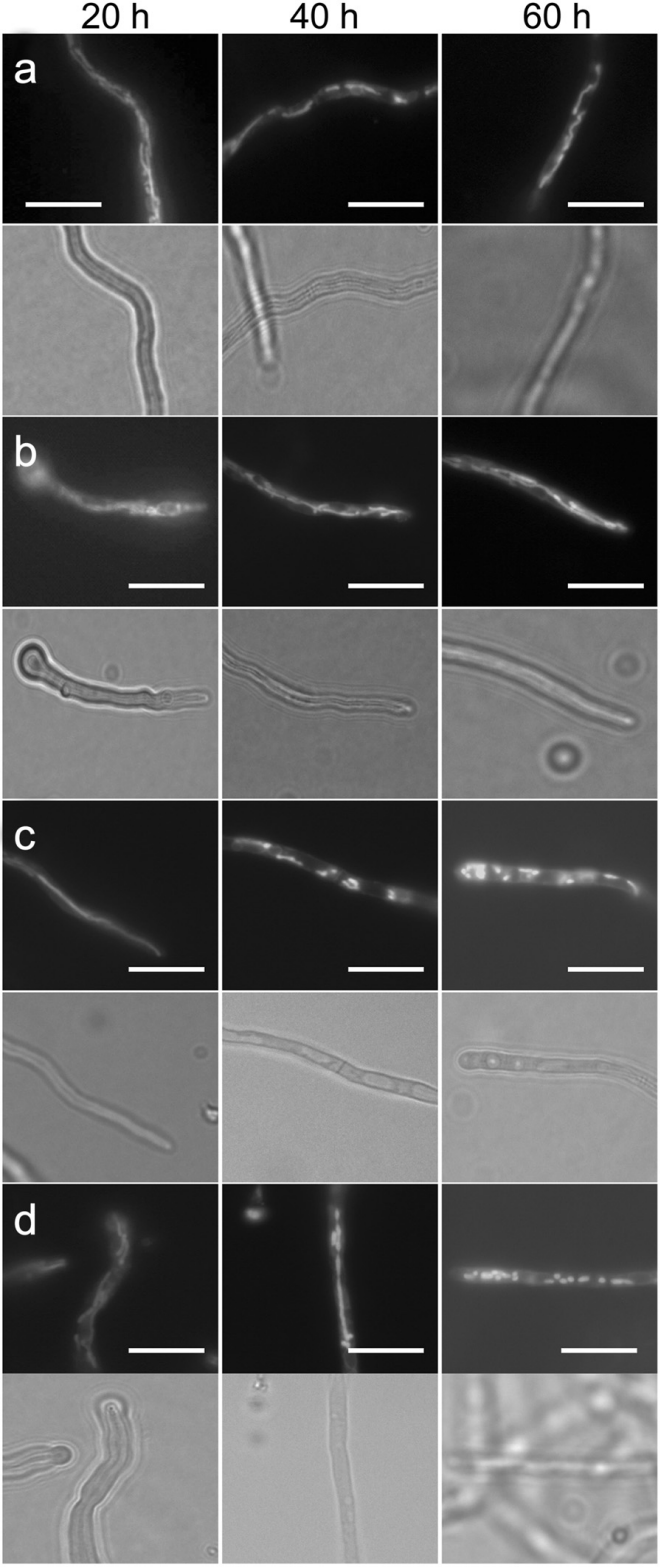
Analysis of mitochondrial morphology in Ws54-1255 treated with methylglyoxal and/or rapamycin. At the indicated times cultures grown on starvation pads supplemented with or without methylglyoxal and/or rapamycin were overlaid with a Mitotracker Green FM solution and analysed using fluorescence microscopy. Representative images are shown for each time point. **a** control; **b** + 0.05 % (v/v) methylglyoxal; **c** + 1 μM rapamycin; **d** + 0.05 % (v/v) methylglyoxal/+ 1 μM rapamycin. Corresponding bright field areas are shown below each fluorescence channel image. Scale bars: 10 μm

Mitochondria display a strikingly dynamic morphology, ranging from small punctuate units to long thread-like structures. Their function depends on their morphology among other factors. ‘Healthy’ mitochondria, capable of synthesizing sufficient amounts of ATP, usually belong to the filamentous morphotype whereas spherical mitochondria are often an indication for cellular stress and death. It has been shown that mitochondrial morphology can be used as a fungal viability marker [30, 31]. In order to address the question whether methylglyoxal and/or rapamycin affect viability of Ws54-1255 mitochondrial morphotypes were determined (Fig. 4, Table 2). Control cultures displayed a filamentous mitochondrial morphology up to 60 h of growth on the starvation pad, indicating that Ws54-1255 is capable to tolerate this pronounced starvation without the induction of cell death (Fig. 4a; Table 2). Cultures growing in the presence of 0.05 % methylglyoxal exhibit a similar behaviour (Fig. 4b; Table 2). However, when cultures were subjected to 1 μM rapamycin, hyphae contained mostly round/spherical mitochondria after 40 and 60 h of cultivation (Fig. 4c; Table 2). Although not demonstrated experimentally, it is suggested that this phenotype might be due to the induction of autophagy (mitophagy) as it is known that mitophagy is correlated with mitochondrial fragmentation [32]. Interestingly, when both methylglyoxal and rapamycin are applied, mitochondrial morphology is mostly filamentous after 40 h of incubation (Fig. 4d; Table 2). This finding suggests that methylglyoxal is able to counteract rapamycin-mediated fragmentation of mitochondria. However, after 60 h of incubation hyphae contain mostly round (fragmented) mitochondria (Fig. 4d; Table 2).

**Table 2:** Mitochondrial morphotypes in Ws54-1255

	20 h	40 h	60 h
Control	fil	fil	fil
+ MG	fil	fil	fil
+ R	fil	rnd	rnd
+ MG/+ R	fil	fil	rnd

*fil* mostly filamentous mitochondria. rnd mostly round, spherical mitochondria. *MG* methylglyoxal (0.05 % [v/v]). *R* rapamycin (1 μM)

### Response of *P. chrysogenum* to methylglyoxal at the protein level

**Fig. 5:**
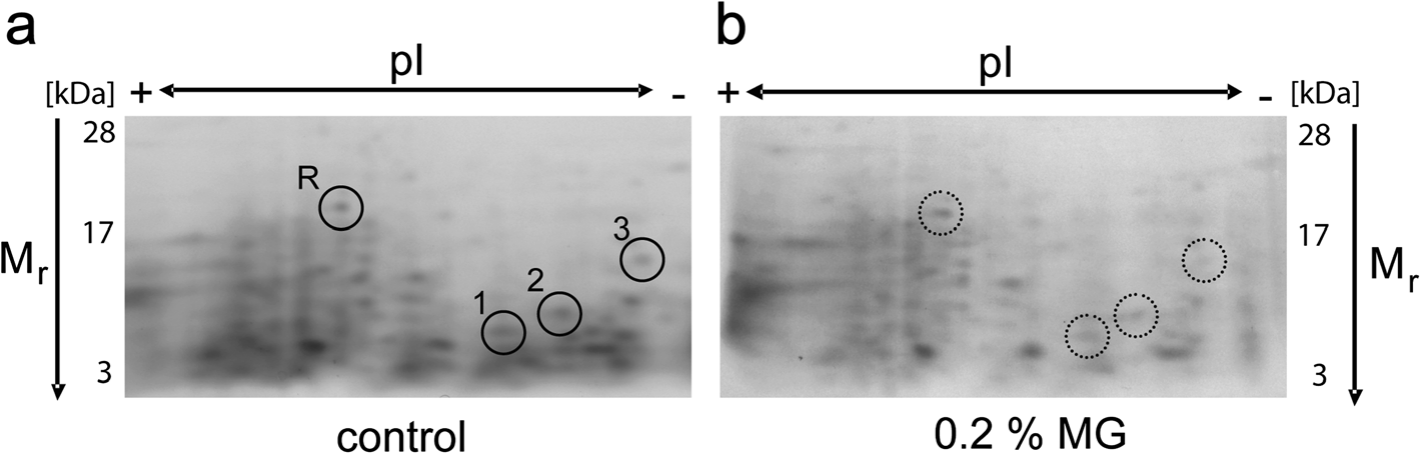
Analysis of changes of protein abundance by methylglyoxal treatment in Ws54-1255 by isoelectric focusing/2D-SDS-PAGE/peptide mass fingerprinting. **a** Separated proteins of an untreated Ws54-1255 culture grown in YGG medium for 2.5 days at 25 °C visualized by Coomassie Blue staining. Proteins with different abundance compared to the culture grown in the presence of 0.2 % (v/v) methylglyoxal are marked by black circles. The identity of selected peptide spots was determined by peptide mass fingerprinting: R-peptide belonging to 60S ribosomal protein L5 (Pc13g11570); 1-peptide belonging to aldo-keto reductase (Pc22g04850); 2-peptide belonging to 5-methyl-tetrahydropteroyl-triglutamate-homocysteine S-methyltransferase (Pc22g18630); 3-peptide belonging to NAD-dependent formate dehydrogenase (Pc12g04310). **b** Separated proteins of a Ws54-1255 culture grown in YGG medium for 2.5 days at 25 °C in the presence of 0.2 % (v/v) methylglyoxal visualized by Coomassie Blue staining. Regions corresponding to the gel shown in panel **a** are marked with dotted circles. pI: isoelectric point, MG: methylglyoxal, M_r_: relative molecular mass

Methylglyoxal is known for its property to modify proteins. It is also known that some proteins are more susceptible for methylglyoxal-mediated glycation than others [5]. The aim of this part of the study was not to detect methylglyoxal-modified proteins but to elucidate the response of *P. chrysogenum* to methylglyoxal present in the growth medium (YGG + 0.2 % [v/v] methylglyoxal). In this complete growth medium as opposed to growth on starvation pads (see above) *P. chrysogenum* was found to tolerate much higher methylglyoxal levels. After cultivation protein extracts from the untreated control and the methylglyoxal-treated sample were prepared and separated using isoelectric focusing and two-dimensional SDS-PAGE. Staining with a highly sensitive Coomassie Blue variant revealed the separated proteins which were in the size range of 3–30 kDa (Fig. 5). A reference protein (Fig. 5, R) was selected that is present (i) in relatively high abundance, (ii) sufficiently isolated from other proteins to facilitate its punching out from the gel for characterisation and (iii) displays approximately the same staining intensity in the two gels (control and methylglyoxal). Three spots that are both isolated from other proteins to minimize the risk of ambiguous identification and present in higher abundance in the control gel compared to the gel containing the separated proteins from the methylglyoxal-treated culture were selected and punched out. Peptide mass fingerprinting revealed the identity of these proteins as follows: Reference protein - peptide from the 60S ribosomal protein L5 (systematic identifier: Pc13g11570); #1 - peptide from aldo-keto reductase (Pc22g04850, EC 1.1.1.2); #2 - peptide from 5-methyl-tetrahydropteroyl-triglutamate-homocysteine S-methyltransferase (Pc22g18630, EC 2.1.1.14) and #3 - peptide from NAD-dependent formate dehydrogenase (Pc12g04310, EC 1.2.1.2). Two of the identified peptides (#1 and #3) belong to proteins that may play a direct role in α-oxoaldehyde metabolism while #2 is involved in homocysteine/methionine metabolism (see Discussion).

## Discussion

The results shown here suggest that methylglyoxal reduces growth of *P. chrysogenum* so that the onset of the stationary phase is delayed. Degradation of peroxisomes by autophagy in the presence or absence of methylglyoxal was utilized to monitor entry into stationary phase. It is widely known that methylglyoxal is capable to readily react with proteins. Are peroxisomal proteins subjected to glycation? In *Arabidopsis thaliana* it was shown that a glyoxalase I homolog, GLX1, is present in peroxisomes [33]. This result hints that there is a need for the removal of methylglyoxal at least in plant peroxisomes (although a GLX2 activity has so far not been demonstrated in plant peroxisomes). It should be kept in mind that the glyoxalase system is not the only line of defense against the toxic effects of α-oxoaldehydes. In addition to the aldehyde reductases and dehydrogenases described in the introduction there are also more indirect mechanisms to prevent the formation of methylglyoxal. In rice it was shown that an increase in methylglyoxal levels correlated with an elevated formation of the glycolysis enzyme triose phosphate isomerase [34]. This reaction results in reduced formation of DHAP which is giving rise to methylglyoxal by spontaneous phosphate elimination (see introduction). So far it is not known if a similar mechanism exists in other organisms but the possibility is certainly intriguing.

Interestingly, methylglyoxal was found to stimulate autophagy in bovine aortic endothelial cells [35]. The authors found increased levels of the autophagy marker proteins Beclin-1 and LC3B-II after methylglyoxal treatment. Furthermore, autophagy flux, as indicated by the appearance of GFP-LC3 dots, was significantly increased in the methylglyoxal-treated cells. It is not known whether other mammalian cell types respond to methylglyoxal in a comparable manner by up-regulating the molecular autophagy machinery.

No candidates of the molecular machinery regulating autophagy were identified in the IEF/2D-SDS-PAGE experiment. However, peptides from three proteins were revealed that are present in reduced abundance when the fungus is grown in medium containing methylglyoxal. Aldo-keto reductase (Pc22g04850) and the NAD-dependent formate dehydrogenase (Pc12g04310) have so far not been characterized in detail in *P. chrysogenum*. It is certainly possible that both proteins are involved in methylglyoxal metabolism/detoxification. NAD-dependent formate dehydrogenase from the methylotrophic yeast *Candida boidinii* was shown to accept methylglyoxal as a substrate in addition to formaldehyde [36]. Furthermore, AKR4C14, a rice aldo-keto reductase, from Thai Jasmine rice is capable to metabolise methylglyoxal in addition to sugars and the aldehydes glutaraldehyde and trans-2-hexenal [37]. Therefore Pc22g04850 and Pc12g04310 might constitute components of a cellular defense system that supports the glyoxalase system in keeping cellular levels of methylglyoxal low. The observation that these proteins are actually present in decreased abundance when *P. chrysogenum* is subjected to methylglyoxal could be explained by a ‘methylglyoxal overload effect’: the proteins are extensively modified by their reaction with methylglyoxal so that they become damaged and are subsequently degraded. Due to the rather high methylglyoxal concentration applied in the experiment the cell fails to maintain a sufficient supply of these enzymes. This would make the corresponding genes prime candidates for their overexpression to further improve the defense capacity of *P. chrysogenum* against methylglyoxal and related α-oxoaldehydes with possibly beneficial effects on biosynthetic productivity. The protein analysis also identified 5-methyl-tetrahydropteroyl-triglutamate-homocysteine S-methyltransferase (Pc22g18630) as a protein which is present in lowered amounts when methylglyoxal is added to the growth medium of *P. chrysogenum*. The enzyme catalyses the final step in methionine biosynthesis (5-methyltetrahydropteroyltri-l-glutamate + l-homocysteine - > tetrahydropteroyltri-l-glutamate + l-methionine) [38]. This reaction is also important for keeping cellular levels of homocysteine low. Homocysteine has several deleterious properties if its cellular levels are elevated, among them apoptosis induction, increased formation of toxic reactive oxygen species and interference with cellular signalling (e. g., due to DNA and RNA methylation, etc. [39]). Therefore it is possible that cellular stress caused by increased methylglyoxal levels and a decrease of 5-methyl-tetrahydropteroyl-triglutamate-homocysteine S-methyltransferase results in the amplification of deleterious signals which could lead to severe cellular defects. For example, the observed inhibitory effect of methylglyoxal on peroxisome degradation could also be based on its indirect effects on other metabolic pathways (i. e., homocysteine metabolism).

## Conclusions

Methylglyoxal reduces growth of *P. chrysogenum* cultivated on starvation pads so that the onset of stationary phase (as monitored by the degradation of GFP-SKL labelled peroxisomes via autophagy) is delayed. Mitochondrial morphology is not compromised by methylglyoxal: the thread-like morphology is not changed and mitochondrial fragmentation is not observed. Three proteins were identified that are present in lower abundance when methylglyoxal is added to a liquid culture of *P. chrysogenum*: #1-Aldo-keto reductase (Pc22g04850); #2–5-methyl-tetrahydropteroyl-triglutamate-homocysteine S-methyltransferase (Pc22g18630) and #3–NAD-dependent formate dehydrogenase (Pc12g04310).

## Methods

### Strains and cultivation conditions

*Penicillium chrysogenum* Wisconsin(Ws)54–1255 (GFP-SKL) [40] was used for growth experiments and for the localisation of green fluorescent protein targeted to peroxisomes (GFP-SKL). Stocks grown on rice were stored at room temperature (RT) in the dark. For initiating growth from stocks and for the preparation of fresh stocks 2–3 rice grains were placed on YGG agar (10 g/l KCl, 20 g/l glucose, 10 g/l yeast nitrogen base, 5 g/l K_2_HPO_4_, 20 g/l yeast extract, 20 g/l agar-agar) and incubated for 4 to 5 days at RT. For cultivation in liquid medium 15–20 rice grains covered with mycelium were inoculated in 50 ml YGG medium in a plastic tube for 1 h with occasional mixing. Afterwards the culture (without the rice grains) was transferred to a 250 ml Erlenmeyer piston. Cultures were incubated for 2 to 3 days at 25 °C at 180 rpm in an Ecotron shaker (Infors GmbH, Einsbach, Germany). As an autophagy negative control for the assessment of degradation of peroxisomes the *P. chrysogenum* strain Δ*atg1* (GFP-SKL) [29] was used in which all autophagy pathways are blocked. Methylglyoxal (cat. no. M0252, Sigma-Aldrich, Taufkirchen, Germany) was added to the media as specified in the corresponding subsections.

### Microscopy

#### *Cultivation of* P. chrysogenum *for microscopy*

For cultivating the Ws54–1255 (GFP-SKL) strain 2–3 rice grains covered with sporulating mycelium (‘green rice’) were incubated in 500 μl YGG medium in a 1.5 ml microcentrifuge tube for 1 d at RT after vortexing the tube for 30 s. From this spore suspension a 1/50 dilution in sterile tap water was prepared. 5 μl of this diluted suspension were pipetted onto an agarose ‘starvation’ pad (1 % (w/v) agarose in sterile tap water containing no additives (control) or 0.05 % (v/v) methylglyoxal and/or 1 μM rapamycin) that was mounted on an object carrier with a central cavity [25]. Samples were incubated at RT for up to 60 h in a wet chamber to prevent desiccation.

For cultivation of the Δ*atg1* (GFP-SKL) strain that has a reduced ability to sporulate [29] 5 μl of an undiluted spore suspension was pipetted onto an YGG agar plate and incubated for 5 d at RT. This resulted in the growth of a mycelium colony. Small pieces of mycelium were transferred with a sterile toothpick into a 1.5 ml microcentrifuge tube containing 400 μl sterile tap water and approx. 100 mg glass beads (0.25 mm-0.5 mm diameter, Carl Roth GmbH, Karlsruhe, Germany). This suspension was vortexed for 2 min. The treatment resulted in fragmentation of the mycelium. 5 μl of the undiluted suspension was pipetted onto a starvation pad and incubated as described above.

#### Microscopy analysis

Samples were analysed with an Axio Imager.M2 fluorescence microscope equipped with an AxioCam Mrc5 digital camera (Carl Zeiss GmbH, Goettingen, Germany). Images documenting mycelium growth (bright-field microscopy) were processed to improve visibility of hyphae using Photoshop 5.0 (Adobe, San José, CA) as follows: (i) inverting the picture (pos > neg), (ii) adjustment of brightness/contrast until hyphae were well resolved. The same settings were applied to all images to allow comparison.

For studying mitochondrial morphology mycelia grown on starvation pads were carefully overlaid with 10 μM Mitotracker Green FM (cat. no. M-7514, Fisher Scientific, Schwerte, Germany) solution for 10 min before analysis by fluorescence microscopy.

### Protein extraction

*P. chrysogenum* cultures were grown for 2.5 d at 25 °C (180 rpm) in YGG medium (either in the absence or presence of 0.2 % [v/v] methylglyoxal). After filtration the mycelium was briefly washed by rinsing it with sterile water. A flat paste of mycelium was wrapped in aluminium foil and stored overnight in a freezer at −25 °C. The next day the frozen mycelium was quickly ground to a fine powder in a chilled mortar containing cold glass beads (0.25 mm-0.5 mm diameter, Carl Roth GmbH, Karlsruhe, Germany) to prevent thawing. The ground powder was stirred in cold isoelectric focusing buffer (8 M urea, 2 % [w/v] CHAPS, 0.5 % [v/v] ampholyte solution pH 3–10 [Fisher Scientific, Schwerte, Germany], 1/1000 protease inhibitor cocktail [cat. no. P8215, Sigma-Aldrich, Taufkirchen, Germany]) at 4 °C for 1 h. After centrifugation (10 min, 6000 x g, 4 °C) the supernatant was carefully removed and an aliquot was analysed for its protein concentration. To the rest of the extract bromophenolblue (final concentration: 0.002 % [w/v]) and 1,4-dithio-DL-threitol (DTT) (final concentration: 20 mM) were added. Care was taken that protein extracts were always thawed on ice and subsequently kept chilled when removed from the freezer.

### Protein concentration determination

Preparations were assayed for the protein concentration by using the ‘Quantipro BCA assay kit’ (Sigma Aldrich, Taufkirchen, Germany) according to the manufacturer’s instructions. An aliquot of the extract was diluted 1/10 in isoelectric focusing buffer (without bromophenolblue and DTT due to the interference of these compounds with the BCA assay). Samples were incubated for 1 h at 60 °C before absorbance was read at 562 nm. Values were compared to a reference curve prepared with different dilutions of a bovine serum albumin solution (supplied with the kit).

### Isoelectric focusing/2D-SDS-PAGE

For isoelectric focusing and 2D-SDS-PAGE the ZOOM system (Fisher Scientific, Schwerte, Germany) was used according to the manufacturer’s instructions. Isoelectric focusing buffer (containing 500 μg *P. chrysogenum* protein extract) was loaded onto pH 3–10 NL (non-linear) IEF strips (Fisher Scientific, Schwerte, Germany) and incubated at 4 °C overnight for rehydration of the polyacrylamide gel on the strips. IEF was performed with the following voltage settings: 1 h 100 V, 2 h 200 V, 3 h 500 V. After the run strips were incubated for 15 min in 1 x NuPAGE LDS sample buffer (Fisher Scientific, Schwerte, Germany) containing 1/10 NuPAGE sample reducing agent (Fisher Scientific, Schwerte, Germany) on a shaker at RT. A similar incubation step for 15 min in 1 x NuPAGE LDS sample buffer containing 125 mM iodoacetamide followed. Strips were then slid onto ZOOM gels (4–12 % polyacrylamide gradient gels, Fisher Scientific, Schwerte, Germany) and immobilised with a pre-warmed solution containing 0.5 % (w/v) agarose in 1 x NuPAGE MES running buffer (Fisher Scientific, Schwerte, Germany). 2D-SDS-PAGE was performed for 45 min at 200 V in 1 x NuPAGE MES running buffer.

### Staining and documentation of polyacrylamide gels

For the detection of separated proteins in polyacrylamide gels the ‘Colloidal Blue staining kit’ (Fisher Scientific, Schwerte, Germany) was used according to the manufacturer’s instructions with the following alterations: gels were incubated overnight in staining solution and de-stained in pure water (changed every two hours) for at least 6 h. For documentation purposes gels were placed on an illumination box (LP200, Doerr, Neu-Ulm, Germany) equipped with a red light filter and photographed using a digital camera.

### Protein identification

Proteins spots of interest were punched out from stained SDS-PAGE gels by using cut pipette tips and analysed by mass spectrometry (MALDI-TOF peptide mass fingerprinting) by Alphalyse A/S, Odense, Denmark. For protein identification the NCBInr database was accessed using Mascot version 2.4. Peptide ions were considered to be positively identified when their Mascot score was above the 95 % confidence level (i. e., > = 90 for the NCBInr database).

## Abbreviations

AGE: Advanced glycation end-product
DHAP: Dihydroxyacetone phosphate
DTT: 1,4-dithio-DL-threitol
GFP: Green fluorescent protein
GLO1: Glyoxalase I
GLO2: Glyoxalase II
IAT: Isopenicillin-N acyltransferase
MG: Methylglyoxal
YGG: Yeast glucose growth
